# LysSAP26, a New Recombinant Phage Endolysin with a Broad Spectrum Antibacterial Activity

**DOI:** 10.3390/v12111340

**Published:** 2020-11-23

**Authors:** Shukho Kim, Jong-Sook Jin, Yoon-Jung Choi, Jungmin Kim

**Affiliations:** Department of Microbiology, School of Medicine, Kyungpook National University, Daegu 41944, Korea; shukhokim@knu.ac.kr (S.K.); in75724@hanmail.net (J.-S.J.); sk8727@daum.net (Y.-J.C.)

**Keywords:** endolysin, multidrug-resistant bacteria, antimicrobial activity

## Abstract

Multidrug-resistant (MDR) bacteria are a major threat to public health. Bacteriophage endolysins (lysins) are a promising alternative treatment to traditional antibiotics. However, the lysins currently under development are still underestimated. Herein, we cloned the lysin from the SAP-26 bacteriophage genome. The recombinant LysSAP26 protein inhibited the growth of carbapenem-resistant *Acinetobacter baumannii*, *Escherichia coli*, *Klebsiella pneumoniae*, and *Pseudomonas aeruginosa*, oxacillin-resistant *Staphylococcus aureus*, and vancomycin-resistant *Enterococcus faecium* with minimum inhibitory concentrations of 5~80 µg/mL. In animal experiments, mice infected with *A. baumannii* were protected by LysSAP26, with a 40% survival rate. Transmission electron microscopy analysis confirmed that LysSAP26 treatment resulted in the destruction of bacterial cell walls. LysSAP26 is a new endolysin that can be applied to treat MDR *A. baumannii*, *E. faecium*, *S. aureus*, *K. pneumoniae*, *P. aeruginosa*, and *E. coli* infections, targeting both Gram-positive and Gram-negative bacteria.

## 1. Introduction

Since the introduction of penicillin, many antimicrobial drugs have been developed and widely used against pathogens, but bacteria have rapidly developed resistance to drugs [1]. The World Health Organization warns that in the 21st century, more people will die from refractory multidrug-resistant (MDR) bacterial infections than from cancer [2]. The decline in antibiotic efficacy against pathogenic bacteria has created an urgent need to find potential alternative antibiotics [1,2].

Recently, endolysin (lysin) has emerged as a promising lead for development as a next-generation antibiotic [3,4]. Lysin is a bactericidal enzyme that bacteriophages (phages) use to penetrate bacteria during infection and also to escape from bacteria after proliferation [5]. The bacteriophage lysin breaks down the peptidoglycan layer constituting the bacterial cell wall. Lysin has several advantages over traditional antibacterial drugs: (1) it has a completely different mechanism of action to conventional antibiotics, (2) it is expected to be advantageous in terms of safety as it has a very high selectivity for bacteria [5], (3) lysin also acts on bacteria in the state of biofilm formation [6], and (4) it is expected to have a low probability of developing resistant bacteria due to its high rate of action [7].

Lysins frequently show modular architecture, combining catalytic domain(s) with or without a cell wall-binding domain that corresponds to host specificity [8]. However, Gram-negative bacteria have an outer membrane covering the peptidoglycan layer, which make them more resistant to the external treatments of lysins than Gram-positive bacteria [9].

Previously, we identified the induced phage SAP-26 and reported that it removed the *Staphylococcus aureus* biofilm when treated with the antibiotic rifampicin. The SAP-26 phage belongs to the Siphoviridae family and is specific to *S. aureus* [10]. In this study, a gene encoding lysin was identified in the SAP-26 phage genome and cloned into a protein expression vector. We named the expressed recombinant protein LysSAP26. The LysSAP26 lysin was tested for its antibacterial activity with several species of MDR bacteria, including *Enterococcus faecium*, *S. aureus*, *Klebsiella pneumoniae*, *Acinetobacter baumannii*, *Pseudomonas aeruginosa*, and *Escherichia coli*. In addition to the in vitro tests, a mouse model infected with *A. baumannii* was used to test the in vivo antibacterial activity of the LysSAP26.

## 2. Materials and Methods

### 2.1. Bacterial Strains and Culture Conditions

The following MDR clinical isolates were used in this study: *E. faecium* (20 isolates), *S. aureus* (20 isolates), *K. pneumoniae* (20 isolates), *A. baumannii* (19 isolates), *P. aeruginosa* (20 isolates), and *E. coli* (20 isolates). The clinical isolates were obtained from the Kyungpook National University Hospital Culture Collection for Pathogens (KNUHCCP) and reference strains were purchased from the Korean Collection for Type Cultures (KCTC) and the American Type Culture Collection (ATCC). Bacterial identification of the clinical isolates was determined by *16S rRNA* sequencing analysis and their antibiotic susceptibility patterns were determined using the VITEK2 system. The sequence types (STs) of *A. baumannii* were analyzed using the OXFORD multi-locus sequence typing (MLST) scheme used in our previous study [11,12]. Liquid broth or solid agar medium, consisting of Luria Bertani (LB) or Mueller Hinton broth (MHB), was used for the growth of bacteria. LB containing 15% glycerol (*v/v*) was used for long-term storage of the bacterial strains. All bacterial strains were cultured at 35 °C aerobically and maintained at −72 °C for long-term storage.

### 2.2. LysSAP26 Sequence In Silico Analysis

The gene encoding *LysSAP26* was identified from the full genome sequence of bacteriophage SAP-26 reported previously (GenBank accession number GU477322; annotated as an amidase) [10]. Similarity analysis between LysSAP26 and other amidases was performed using the National Center for Biotechnology Information (NCBI) protein Basic Local Alignment Search (BLAST) search and the constraint-based multiple alignment tool.

### 2.3. Construction of the LysSAP26 Expression Vector

The open reading frame encoding *LysSAP26* was amplified by polymerase chain reaction (PCR) using the primers *Nde*I-saplys (5′-GGGAATTCCATATGAAAACATACAGTGAAGC-3′) and *Xho*I-saplys (5′-ATCCGCTCGAGAAACACTTCTTTCACAATC-3′). The amplified PCR product (775 bp) was purified and digested with *Nde*I and *Xho*I restriction enzymes (target sequences are underlined in each primer) and ligated into the pET21a expression vector using the Gibson Assembly^®^ kit (New England Biolabs, Beverly, MA, USA). The ligation product was introduced into *E. coli* DH5α, and the transformants were selected on LB agar plates containing ampicillin (100 μg/mL). The resulting plasmid, pLysSAP26, which facilitated the expression of recombinant LysSAP26 with a C-terminal 6-histidine tag (-LEHHHHHH-C-terminal) under control of the T7 promoter, was purified and verified by sequencing analysis.

### 2.4. Purification of LysSAP26

The procedure for purification of the LysSAP26 protein was the same as in our previous report [4]. The *E. coli* BL21 (DE3) strain was transformed with pLysSAP26, and following protein expression induction with IPTG, the His-tagged LysSAP26 recombinant protein (30.1 kDa) was purified by Fast Protein Liquid Chromatography (FPLC) affinity chromatography. The final purified LysSAP26 was dialyzed and contained using the lysis buffer (50 mM Tris–HCl (pH 8.0), 200 mM NaCl, and 100 mM ZnCl_2_). Sodium dodecyl sulfate–polyacrylamide gel electrophoresis, protein quantification, and Western blot analysis were performed as described in [4].

### 2.5. Antibacterial Activity Test of LysSAP26 against Various Clinical Isolates

To determine if LysSAP26 has antimicrobial activity against six species of bacteria (*E. faecium*, *S. aureus*, *K. pneumoniae*, *A. baumannii*, *P. aeruginosa* and *E. coli*), a microdilution method was performed as described in [4,13]. The concentration of LysSAP26 for the minimal inhibitory concentration (MIC) and minimal bactericidal concentration (MBC) measurements ranged from 1.25 to 80.0 μg/mL. The time–kill assay for LysSAP26 with *A. baumannii* ATCC 17978 was performed by preparing a bacterial suspension as for the microdilution method, adding LysSAP26 to a final concentration of 10 µg/mL. The sampling time points were 0, 2, 4, 6, 8, and 24 h from the start of incubation, and the samples were diluted and spread onto LB plates to determine the colony-forming units (CFUs). For the negative control, buffer was added instead of LysSAP26. Two independent replicates were performed.

### 2.6. Transmission Electron Microscopic (TEM) Analysis of A. baumannii Treated with LysSAP26

The *A. baumannii* ATCC 17978 strain was cultured in LB broth and harvested in the log phase of growth (OD600 = 0.5). Bacterial cells were placed into a 96-well plate (10^4^ CFUs/well) and purified LysSAP26 (10 μg/mL) or buffer only (50 mM Tris-HCl and 500 mM NaCl) was added. The final volume of each well was adjusted to 200 μL with MHB. After 5 h of incubation at 37 °C, the LysSAP26-treated and buffer-treated (negative control) *A. baumannii* ATCC 17978 were placed on a carbon-coated copper grid. The samples were negatively stained with 2% uranyl acetate for 50 s and examined by transmission electron microscopy (TEM; Hitachi-7000; Hitachi, Tokyo, Japan) at 60 kV [4].

### 2.7. LysSAP26 Protection Assay with A. baumannii Systemic Infection Mouse Model

For the protection assay, female neutropenic BALB/c mice (5 weeks old, 16–19 g) and the *A. baumannii* ATCC 17978 strain were used. The protection assay was performed as described in [4]. Systemic infection was induced by the intraperitoneal injection of 200 μL of bacterial inoculum (1 × 10^9^ CFUs) harvested in the log-phase of growth. The mice were divided into 6 groups as follows (5 mice/group):

- Group 1. A negative control group injected with 100 μL of the lysis buffer alone;
- Group 2. First safety test group injected with 35 μg of LysSAP26 alone;
- Group 3. Second safety test group injected with 70 μg of LysSAP26 alone;
- Group 4. An infection control group infected with *A. baumannii* ATCC 17978;
- Group 5. First lysin-treated group infected with *A. baumannii* ATCC 17978 and treated with 35 μg of LysSAP26 30 min after infection;
- Group 6. Second lysin-treated group infected with *A. baumannii* ATCC 17978 and treated with 70 μg of LysSAP26 30 min after infection.

The mice were monitored every day for 6 days following infection. All animal experimental procedures were approved by the Animal Care Committee of Kyungpook National University.

## 3. Results

### 3.1. LysSAP26 DNA and Protein Sequence Analysis

**Figure 1:**
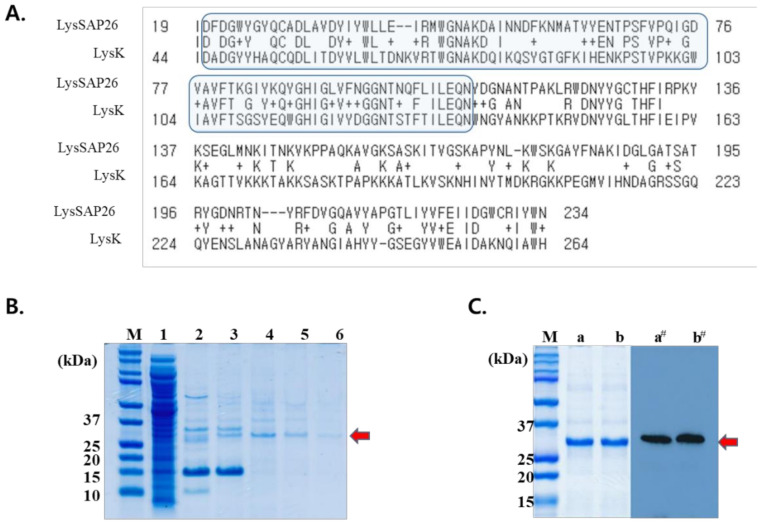
In silico analysis and purification of LysSAP26. (**A**) Two amino acid sequence alignments between LysSAP26 and LysK using NCBI protein BLAST. Highlighted area: cysteine, the histidine-dependent amidohydrolase/peptidase (CHAP) domain. Middle line reports that letters are an identical match, + is a positive match, space is a match with zero, and dashes are gaps. (**B**) Protein analysis by sodium dodecyl sulfate–polyacrylamide gel electrophoresis and (**C**) Western blot analysis of LysSAP26. The red arrow indicates the 30.1 kDa LysSAP26 endolysin with a 6-histidine tag. M: molecular size marker, lane 1: flow-through sample, lane 2–6: eluted fractions from Fast Protein Liquid Chromatography (FPLC) wash sample, lanes a and b: purified LysSAP26, lanes a’ and b’: blotted lane a and b proteins showing Western signals with an anti-His monoclonal antibody.

The LysSAP26 protein is encoded by the 26th open reading frame of the SAP26 phage and consists of 251 amino acids (AAs; 29.1 kDa). The protein BLAST search results showed that the LysSAP26 aligns with the N-acetylmuramoyl-L-alanine amidase, autolysin, and cysteine, the histidine-dependent amidohydrolase/peptidase (CHAP) domain-containing protein of *S. aureus* (E-value: 0.0). The LysSAP26 sequence showed homology with the CHAP domain (from 20th to 109th AA, E-value: 3.85 × 10^−8^) [14]. The LysSAP26 protein sequence (from 19th to 234th AA) also showed similarity with LysK (E-value: 8 × 10^−4^), a well-known endolysin of *S. aureus* phage K (Figure 1A), and with LysSA11 amidase (from 3rd to 139th AA of LysSAP26, E-value: 3 × 10^−16^) of *S. aureus* phage SA11 [14,15]. However, the C-terminal domain of LysSAP26 (from 140th to 251st AA) did not show any high similarity results with the reported proteins or domains.

### 3.2. LysSAP26 Purification

The quantitative amount of LysSAP26 protein purified from a 1 L culture was approximately 16 mg, and the purity of the protein was more than 99%, according to the densitometry band intensity analysis by SDS-PAGE (Figure 1B). Western blot analysis using an anti-His monoclonal antibody showed an approximately 30 kDa signal that suggested the purified protein was the His-tagged LysSAP26 recombinant protein (Figure 1C).

### 3.3. Antibacterial Activity of LysSAP26

The antibacterial activities of the purified LysSAP26 against six representative bacterial strains are shown in Table 1. Treatment with LysSAP26 inhibited the growth of all tested bacterial species. The MIC for *S. aureus* ATCC 33591 was 40 µg/mL, while the MIC of *K. pneumoniae* KCTC 2208, *P. aeruginosa* PAO1, and *E. coli* ATCC 25922 were all 20 µg/mL. The MIC for both *A. baumannii* ATCC 17978 and ATCC 19606 was 10 µg/mL, which was the lowest MIC value among the five bacterial species (Table 1). The MBC values for *A. baumannii* and *P. aeruginosa* were 10 µg/mL and 20 µg/mL, respectively, which were identical to the MIC values. The MBC values of the other three bacterial species were over 80 µg/mL (Table 1).

To further examine the effect of LysSAP26 on the clinical MDR strains, clinical isolates, including ESKAPE (*E. faecium*, *S. aureus*, *K. pneumoniae*, *A. baumannii*, *P. aeruginosa*, and *E. coli*) bacteria, were subjected to susceptibility tests, and the results are shown in Table 2. The MIC values of 20 isolates of vancomycin-resistant *E. faecium* (VRE) were 40 µg/mL (18 isolates) and 80 µg/mL (two isolates). The MIC of LysSAP26 for all 20 isolates of oxacillin-resistant *S. aureus* (ORSA) was 20 µg/mL. The MIC of LysSAP26 for all 20 isolates of carbapenem-resistant *K. pneumoniae* (CRKP) was 40 µg/mL. The MIC value for 20 carbapenem-resistant *P. aeruginosa* (CRPA) isolates was 40 µg/mL, except for one isolate having a MIC of 20 µg/mL. The MIC values for 20 carbapenem-resistant and/or cephalosporin-resistant *E. coli* isolates were 20 µg/mL in five isolates and 40 µg/mL in 15 isolates (Table 2).

**Figure 2:**
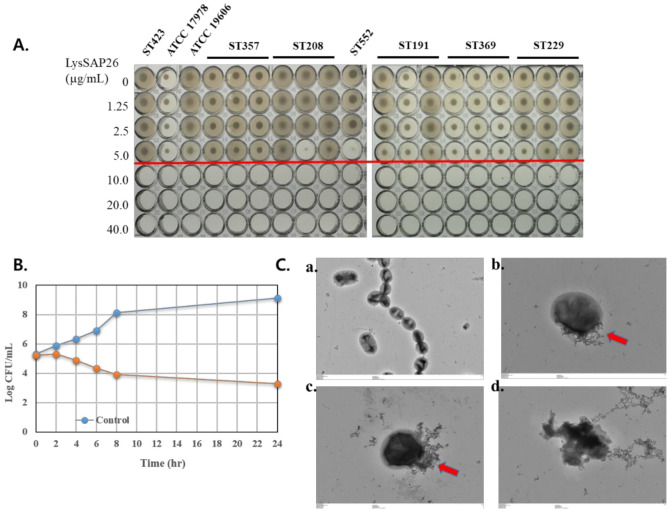
Antibacterial activity of LysSAP26. (**A**) Antimicrobial activity (MIC) test results of LysSAP26 against two type strains and 17 carbapenem-resistant *A. baumannii* (CRAB) isolates. Seven different sequence types (STs) were present. The red line indicates the break points of the MICs. (**B**) Time–kill assay of LysSAP26 against *A. baumannii* ATCC 17978. LysSAP26 was added to a final concentration of 10 µg/mL into the bacterial suspensions (10^5^ colony forming units (CFUs)/mL in Mueller Hinton broth (MHB)). The samples were taken at 0, 2, 4, 6, 8, and 24 h from the start of incubation, diluted and spread onto Luria Bertani (LB) plates to determine the CFUs. Control was the endolysin buffer addition instead of LysSAP26. This experiment was performed three times independently. The error bars are invisible even though those were inserted. (**C**) Representative transmission electron microscopy images of the untreated control (**a**) and LysSAP26 (10 μg/mL)-treated *A. baumannii* ATCC 17978 (**b**–**d**). The *A. baumannii* ATCC 17978 at the exponential phase was treated with LysSAP26 at 37 °C for 5 h and the samples were prepared for the observation as described. Panels (**b**) and (**c**) show perforated cell membrane (arrows) and panel (**d**) shows the complete disorder of a cell. Panel (**a**) is a negative control treated with buffer only.

Treatment with LysSAP26 inhibited the growth of all 17 isolates tested for carbapenem-resistant *A. baumannii* (CRAB), which harbored seven different STs (three of ST191, ST208, ST229, ST357, and ST369, and one of ST423 and ST552) (Figure 2A). All 17 isolates had an MIC value of 10 µg/mL. The MBC values were identical to the MIC values in all 17 isolates (Table 2).

The antibacterial activity of LysSAP26 against *A. baumannii* ATCC 17978 was followed by the time–kill assay using 10 µg/mL of LysSAP26. As shown in Figure 2B, the number of CFUs of *A. baumannii* decreased after 2 h. After 24 h incubation with 10 µg/mL, the LysSAP26, bacterial number was decreased by two log-fold compared to the initial number of bacteria.

### 3.4. A. baumannii Cell Features Affected by LysSAP26

The effect of LysSAP26 on *A. baumannii* was examined by TEM (Figure 2C). Treatment of *A. baumannii* with 10 μg/mL of LysSAP26 resulted in few viable bacteria. The cell morphologies of the remaining bacteria looked abnormal and exhibited bacterial cell wall damage Figure 2C(b–d).

### 3.5. In Vivo Protection Assays of LysSAP26

**Figure 3:**
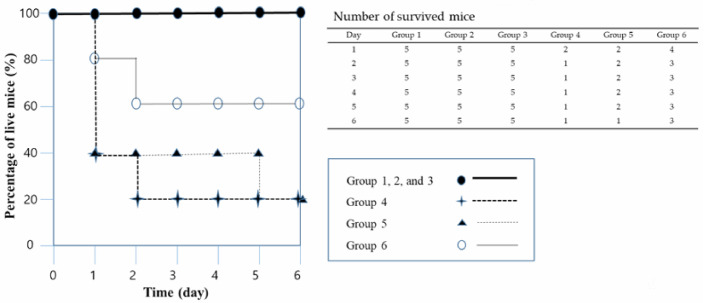
Protection assay results of LysSAP26 using an *A. baumannii* systemic infection mouse model. A mouse mortality graph and a table with raw data are presented together. Group 1: a negative control group (100 μL of the lysis buffer), Group 2: first safety group (35 μg of LysSAP26), Group 3: second safety group (70 μg of LysSAP26), Group 4: an infection control consisting of infected mice without any treatment, Group 5: treatment with 35 μg of LysSAP26, and Group 6: treatment with 70 μg of LysSAP26.

*A. baumannii* was our foremost target bacterium among the MDR pathogenic bacteria tested, so the *A. baumannii* murine systemic infection model was prepared for the assessment of the antibacterial activity of LysSAP26 (Figure 3). Neutropenic mice with intraperitoneally induced *A. baumannii* infection were treated with either LysSAP26 (Groups 5 and 6) or saline (Group 4). In the saline-treated mice (Group 4), three of the five mice died on the first day, and then only one of the five mice survived past the second day of the infection. Mice treated with 35 µg of LysSAP26 (Group 5) had a 40% survival rate until five days after the injection date, while mice treated with 70 µg of LysSAP26 (Group 6) had a 60% survival rate until six days after the injection date. Groups 1–3 did not show any death, indicating that the negative control was normal and that both 35 µg and 70 µg LysSAP26 injections were 100% safe for six days.

## 4. Discussion

Lysin is an enzyme produced by phages that breaks down bacterial cell walls to release phage progeny [16]. When a small amount of recombinant lysin is added externally to Gram-positive bacteria, the cell wall is digested immediately and the target bacteria rapidly die. However, lysin is less effective in killing Gram-negative bacteria due to the presence of an outer membrane in the bacterial cell wall [17]. A lysin that can act on both Gram-positive and Gram-negative bacteria would be a useful antibacterial agent in an era of increasing antibiotic resistance [17].

Previously, we described an induced *S. aureus* phage, SAP-26 [10]. In this study, we cloned and overexpressed LysSAP26, the lysin from the SAP-26 phage. According to in silico DNA analysis, LysSAP26 contains a lysin-harboring CHAP domain, without an amidase or SH3 domain [18]. LysSAP26 shows high similarity with the LysK and LysSA11 amidase, in part. The unaligned C-terminal region of LysSAP26 is a novel sequence that may be a cell wall-binding domain. The activity of the lysin could be enhanced by removing the binding domain [19,20]. We made deletion mutants without the unaligned C-terminal domain and found that the mutants exhibited a similar or better antibacterial activity. Further studies are required to explain this observation. From this point of view, relative experiments should be done in depth.

LysK kills a wide range of pathogenic *Staphylococci* spp., including both human and veterinary strains, but the killing activity is specific to the genus *Staphylococcus* [21]. The LysSA11 endolysin, derived from the virulent *S. aureus* phage SA11, has lytic activity against staphylococcal strains, but does not act on either Gram-positive or Gram-negative bacteria [15]. In this study, LysSAP26 showed growth inhibition of all tested clinical isolates, including CRAB, VRE, methicillin resistant *S. aureus* (MRSA), CRKP, CRPA, and carbapenem-resistant and/or cephalosporin-resistant *E. coli.* Our results suggest that LysSAP26 has an extended antibacterial activity and can target both Gram-positive and Gram-negative bacteria. We define “broad-spectrum activity” as antibacterial activity against both Gram-positive and Gram-negative clinical isolates. This broad antibacterial activity has only been experimentally shown in vitro. For future studies, additional bacterial species for antibacterial activities in vitro as well as the antibacterial pharmacodynamics of LysSAP26 in vivo should be investigated.

LysSAP26 showed the strongest antibacterial activity against *A. baumannii* and the lowest MIC value of LysSAP26 among the tested ESKAPE bacteria. In addition, the MIC and MBC values of LysSAP26 against *A. baumannii* were identical. Similar to *A. baumannii*, the MIC and MBC values of LysSAP26 against *P. aeruginosa* were the same, suggesting the bactericidal efficacy of LysSAP26 against these two bacteria. However, the MBC and MIC values of LysSAP26 for the remaining four bacterial species varied greatly, suggestive of bacteriostatic efficacy against *E. faecium*, *S. aureus*, *K. pneumoniae,* and *E. coli*. These results are similar to those of LysSS, which also showed bactericidal activities against only *A. baumannii* and *P. aeruginosa* [4].

Recently, we described newly generated recombinant peptidoglycan-degrading enzymes, LysSS and Ablysin [4,10]. The antibacterial activity of LysSAP26 against *A. baumannii* isolates was similar to those of LysSS harboring a lysozyme-like domain and Ablysin with GH25 muramidase homology. The three lysins have bactericidal activity against CRAB isolates, with the predominant subtypes of ST357, ST208, ST552, ST191, ST369, and ST229 found in Korea and other countries [12,22]. Among the three lysins, LysSAP26 showed the strongest antibacterial activity. The MIC values for LysSS, Ablysin, and LysSAP26 were 63~250 µg/mL, 110~230 µg/mL, and 5~10 µg/mL, respectively. In TEM analysis, LysSAP26 ruptured or degraded the bacterial cell membrane of *A. baumannii.* LysSAP26 was initially shown to degrade the lateral side of the bacterial cell wall (Figure 2C(b,c)), eventually crushing the cells and killing them (Figure 2C(d)). This is similar to the mechanism of action of LysSS lysin, while the lysozyme-like Ablysin perforates the cell wall, followed by total cell shrinkage [4,10]. Abdelkader et al. reported recently that LysMK34 lysin has turgor pressure-dependent antibacterial activity against *A. baumannii* [23]. We hypothesized that LysSAP26 would penetrate into the outer membrane and degrade peptidoglycan partly, and the damaged bacteria eventually would be lysed by force of turgor pressure. This hypothesis should be investigated using well-defined buffers in a future study.

In a murine systemic infection model, treatment with 35 µg and 70 µg of LysSAP26 resulted in 20% and 60% survival rates, respectively, showing the protection efficacy of LysSAP26 against *A. baumannii*–infected mice in a dose-dependent manner. According to the cytotoxicity test with A549 cells, which are human alveolar basal epithelial cells, there was no critical cytotoxicity as a result of increasing the concentration by 50 ug/mL of LysSAP26 after 24 h of exposure. Future studies are needed to determine the optimal dose of LysSAP26 and to obtain preclinical data (e.g., toxicological data) in various disease models induced by ESKAPE pathogens.

This study reports a new phage lysin, LysSAP26, that inhibits the growth of ESKAPE pathogens in vitro and protects systemic *A. baumannii*-infected mice. Lysins currently being commercialized and under clinical trials actively target Gram-positive pathogens, while effective lysins that target Gram-negative bacteria are limited. LysSAP26 acts on both Gram-positive and Gram-negative bacteria, including CRAB. Therefore, LysSAP26 is a promising candidate for use as a biocontrol tool against MDR ESKAPE pathogens.

## Figures and Tables

**Table 1:** Minimum inhibitory concentrations (MICs) and minimum bactericidal concentrations (MBCs) of LysSAP26 against representative bacterial strains.

Bacterium	Strain	MIC (µg/mL)	MBC (µg/mL)
*Staphylococcus aureus*	ATCC 33591	40	>80
*Klebsiella pneumoniae*	KCTC 2208	20	>80
*Acinetobacter baumannii*	ATCC 17978	10	10
*Acinetobacter baumannii*	ATCC 19606	10	10
*Pseudomonas aeruginosa*	PA01	20	20
*Escherichia coli*	ATCC 25922	20	>80

**Table 2:** Antimicrobial activity of LysSAP26 against drug-resistant clinical isolates.

Bacteria	No. of Isolates Tested	Source ^1^	MIC ^2^ (µg/mL)	MBC ^3^ (µg/mL)
*Enterococcus faecium*	20	KNUHCCP	40–80	>80
*Staphylococcus aureus*	20	KNUHCCP	20	>80
*Klebsiella pneumoniae*	20	KNUHCCP	40	>80
*Acinetobacter baumannii*	17	KNUHCCP	10	10
*Pseudomonas aeruginosa*	20	KNUHCCP	20–40	20–40
*Escherichia coli*	20	KNUHCCP	20–40	>80

^1^ KNUHCCP: Kyungpook National University Hospital Culture Collection for Pathogens, ^2^ MIC: minimum inhibitory concentration, ^3^ MBC: minimum bactericidal concentration.
